# Supplementary figures and images for: AAAKB: A manually curated database for tracking and predicting genes of Abdominal aortic aneurysm (AAA)
Source: PLoS One. 2023 Dec 15;18(12):e0289966. doi: 10.1371/journal.pone.0289966 (PMC10723669; doi:10.1371/journal.pone.0289966)

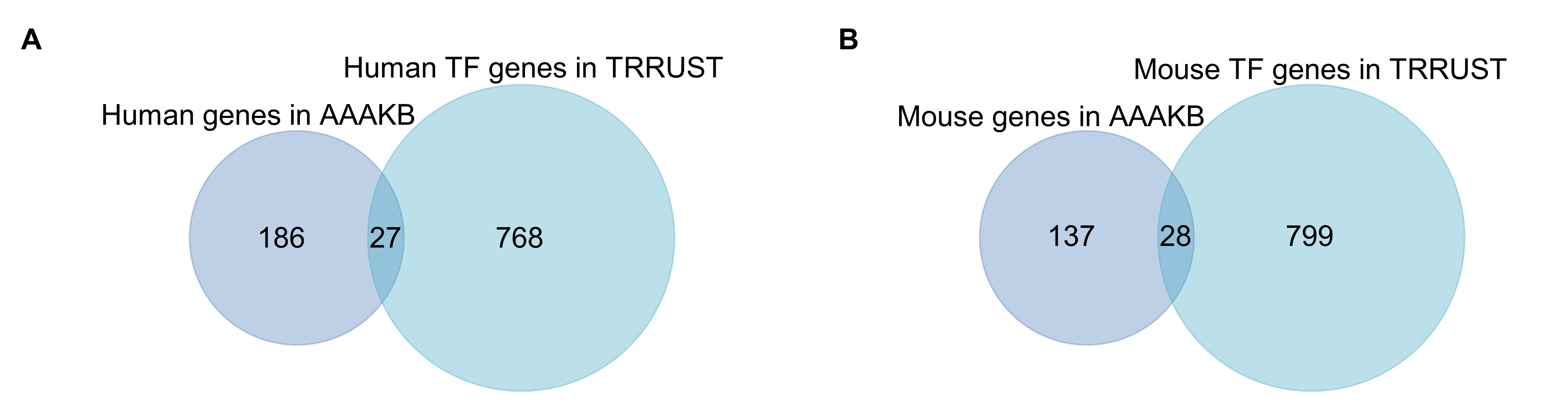

Supplement: S1 Fig — (A, B) The intersections showed that 27 human genes and 28 mouse genes in AAAKB were curated as transcriptional factors (TFs) in the TRRUST database, respectively. (TIF) [file pone.0289966.s001.tif]
