# Supplementary material for: AAAKB: A manually curated database for tracking and predicting genes of Abdominal aortic aneurysm (AAA)
Source: PLoS One. 2023 Dec 15;18(12):e0289966. doi: 10.1371/journal.pone.0289966 (PMC10723669; doi:10.1371/journal.pone.0289966)
Supplement: Attachment — Submitted filename: Response to Reviewers.docx [file pone.0289966.s003.docx]

**Response to Reviewers Comments**

(*Reviewer comments in Times New Roman italics*, responses in Arial)

**We thank the reviewer for his efforts and useful comments that helped us clarify and improve our study and manuscript. We have addressed each comment below and made accompanying changes to the revised manuscript. All changes have been highlighted.**

*- Improve the introductory section of the manuscript by highlighting and listing the “contributions” points of this research and the work's limitations;*

**Response:** Thank you for your valuable comments. We have revised the introductory section of the manuscript to highlight the contributions and limitations of our research on Page 3-4.

*- The study uses the SVM algorithm to predict potentially AAA-associated genes; It is crucial to validate these predictions through more experimental studies, such as gene expression analysis, to confirm their roles in AAA development. Moreover, it is crucial to validate with other ML techniques for comparison (RF,NB,KNN,etc), and in the meantime, describe hyper parameter tuning and settings.*

**Response:** Good point. Following your suggestion, I involved another three ML techniques (RF, NB, KNN) for prediction. And you are right that RF overall performs the best. Therefore, we chose RF as the optimal and final prediction model for predicting potentially AAA-associated genes. We have updated this section of the results on page 8.

Regarding the hyperparameter tuning and settings, we apologize for not providing these details in the original manuscript. In the revised manuscript, we have added sections in methods detailing the ML model parameters used for gene prediction on page 4-5.

We agree that it is important to validate the ML-based predictions through gene expression analysis to confirm their roles in AAA development. In our revised manuscript, we have emphasized this point in the result section on page 8, and we have updated the corresponding method on page 5.

- *The conclusion section needs a clear exposition on how the initial goal of this study was achieved. I suggest this part to support these with adequate numerical results and references, offering a more critical/discursive view of future research.*

**Response:** Thank you for your suggestion. We have revised the conclusion section to better highlight the contributions of our research and the importance of our gene database in advancing AAA research on page 10-11. In this version, we have discuss the potential of our predicted AAA genes as therapeutic targets, and how it could facilitate researchers to better select their focused gene in the sophisticated signaling network.
